# Engineered T Regulatory Type 1 Cells for Clinical Application

**DOI:** 10.3389/fimmu.2018.00233

**Published:** 2018-02-15

**Authors:** Silvia Gregori, Maria Grazia Roncarolo

**Affiliations:** ^1^San Raffaele Telethon Institute for Gene Therapy (SR-Tiget), IRCCS San Raffaele Scientific Institute, Milan, Italy; ^2^Division of Stem Cell Transplantation and Regenerative Medicine, Department of Pediatrics, ISCBRM, Stanford School of Medicine, Stanford, CA, United States

**Keywords:** T regulatory type 1 (Tr1) cells, tolerance, T regulatory cell-based therapy, IL-10, gene transfer

## Abstract

T regulatory cells, a specialized subset of T cells, are key players in modulating antigen (Ag)-specific immune responses *in vivo*. Inducible T regulatory type 1 (Tr1) cells are characterized by the co-expression of CD49b and lymphocyte-activation gene 3 (LAG-3) and the ability to secrete IL-10, TGF-β, and granzyme (Gz) B, in the absence of IL-4 and IL-17. The chief mechanisms by which Tr1 cells control immune responses are secretion of IL-10 and TGF-β and killing of myeloid cells *via* GzB. Tr1 cells, first described in peripheral blood of patients who developed tolerance after HLA-mismatched fetal liver hematopoietic stem cell transplantation, have been proven to modulate inflammatory and effector T cell responses in several immune-mediated diseases. The possibility to generate and expand Tr1 cells *in vitro* in an Ag-specific manner has led to their clinical use as cell therapy in patients. Clinical grade protocols to generate or to enrich and expand Tr1 cell medicinal products have been established. Proof-of-concept clinical trials with Tr1 cell products have demonstrated the safety and the feasibility of this approach and indicated some clinical benefit. In the present review, we provide an overview on protocols established to induce/expand Tr1 cells *in vitro* for clinical application and on results obtained in Tr1 cell-based clinical trials. Moreover, we will discuss a recently developed protocol to efficient convert human CD4^+^ T cells into a homogeneous population of Tr1-like cells by lentiviral vector-mediated IL-10 gene transfer.

## Introduction

T regulatory type 1 (Tr1) cells are a subset of adaptive CD4^+^ T cells that promote immune tolerance and control excessive and/or inappropriate inflammation mediated by effector T cells and antigen-presenting cells (APCs). In contrast to thymic-derived T regulatory cells (Tregs) that constitutively express the transcription factor (TF) FOXP3 (FOXP3^+^ Tregs) (1, 2), Tr1 cells can only transiently upregulate FOXP3 upon activation (3–7).

Tr1 cells are memory CD4^+^ T cells that co-express the integrin alpha2 subunit (CD49b) and the lymphocyte-activation gene 3 (LAG-3) (7). Although other cell surface markers, including PD-1, ICOS, TIGIT, CD39, CD73, TIM-3, GITR, OX40, TNFRSF9, and CEACAM-1 (8), have been associated with Tr1 cells, their expression on other cell types precludes them from being defined as Tr1-specific markers. Tr1 cells produce high levels of IL-10 and TGF-β; variable amounts of IFN-γ; and low/no IL-2, IL-4, and IL-17 (6, 7, 9, 10) and have a specific gene signature (7). In addition, Tr1 cells have unique metabolic requirements that distinguish them from FOXP3^+^ Tregs: Tr1 cells depend on glycolysis and are inhibited by hypoxia and extracellular ATP (11), while peripheral FOXP3^+^ Tregs depend on fatty acid oxidation (12).

**Figure 1:**
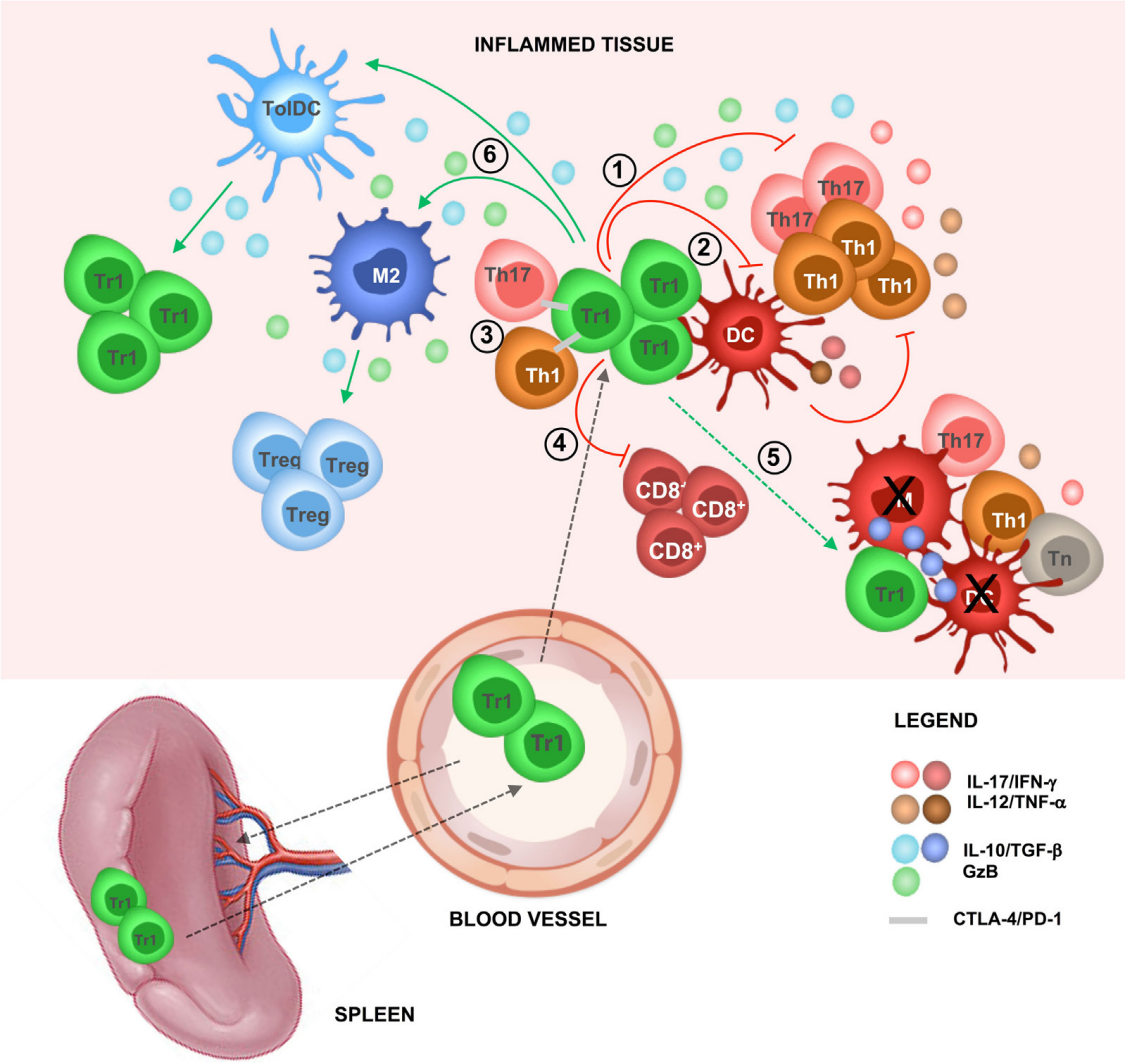
T regulatory type 1 (Tr1)-mediated suppression *in vivo*. In steady-state condition, Tr1 cells reside in the spleen and circulate in the periphery. During inflammation, Tr1 cells are recruited to the site of tissue injury (i.e., after infections, autoimmune reactions, or transplantation) and are activated by professional antigen-presenting cells [APCs; dendritic cells (DCs)] *via* their T cell receptor, thus by their cognate antigen (Ag). Upon activation, Tr1 cells secrete IL-10 and TGF-β and (1) directly inhibit effector T cell (i.e., Th1 and Th17 cells) proliferation and pro-inflammatory cytokines production and (2) indirectly inhibit effector T cells by modulating professional APCs (i.e., downregulation of costimulatory and HLA class II expression and inhibition of pro-inflammatory cytokine secretion). (3) Tr1 cells can suppress effector T cells by cell-to-cell contact-mediated mechanisms, (4) suppress CD8^+^ T cell responses (i.e., proliferation and IFN-γ production), and (5) mediate bystander suppression by specifically killing professional APCs [DC or macrophages (M)], thus preventing naive T (Tn) cell priming and reactivation of effector T cells (i.e., Th1 and Th17 cells). Concomitantly, (6) Tr1 cells *via* IL-10 and TGF-β promote the induction of tolerogenic DC and anti-inflammatory macrophages (M2), which in turn promote *de novo* induction of Tr1 cells and T regulatory cells (Tregs), restoring tissue homeostasis and promoting long-term tolerance.

The main mechanism of Tr1-mediated suppression is the secretion of IL-10 and TGF-β. Importantly, Tr1 cells require activation *via* their T cell receptor, thus by their cognate antigen (Ag), to mediate suppression, but, once activated, they mediate bystander suppression against other Ags (6, 9). The expression of granzyme (Gz) B endows Tr1 cells with the ability to specifically kill myeloid APCs (6, 13). Similar to FOXP3^+^ Tregs, Tr1 cells also inhibit T cell responses *via* CTLA-4/CD80 and PD-1/PDL-1 interactions (14) and metabolic disruption (15) (Figure 1). IL-10 signaling is required for maintaining high IL-10 production by Tr1 cells, which in turn is necessary for controlling inflammatory responses. Notably, in the absence of IL-10-mediated signaling, Tr1 cells lose their ability to secrete IL-10, but they still express GzB and CTLA-4 (16). These findings suggest that in the absence of IL-10/IL-10R-mediated signaling, and consequent IL-10 production, Tr1 cells may suppress immune responses *via* alternative mechanisms such as specific killing of APCs and/or cell-to-cell contact-mediated inhibition of effector T cells and APCs (Figure 1).

IL-10 is the driving cytokine for Tr1 cell differentiation and function (9, 16). In the past years, it has become evident that activation of CD4^+^ T cells in the presence of IL-27, key regulator of IL-10 production in T cells (17), promotes the differentiation of Tr1 cells in mice (11, 18–20). In T cells, the downstream effects of IL-10/IL-10R interaction is signaling *via* STAT3 (21), and although no formal proof for the critical role of STAT3 in Tr1 cell differentiation exists, several evidences indicate that it represents the link between IL-10/IL-10R and downstream activation of TFs involved in Tr1 cell induction and functions. Specifically, (i) overexpression of active STAT3 in T cells promotes Tr1 cell induction (22), (ii) IL-27-dependent induction of IL-10 is STAT1 and STAT3 mediated (23), and (iii) STAT3 interacts with the aryl hydrocarbon receptor (AhR) that by inducing HIF-1α degradation leads to the stabilization of the glycolytic metabolism in Tr1 cells (11).

A plethora of TFs have been shown to be involved in driving Tr1 cell differentiation, phenotype, and functions (24). The TFs c-Maf and AhR induced by IL-27 bind together to transactivate the *IL-21* and *IL-10* promoters. While IL-21 maintains c-Maf and AhR expression, the expression of IL-10 is essential for the suppressive function of Tr1 cells. Moreover, IL-27-induced AhR, alone or with an unknown cofactor, promotes GzB expression in Tr1 cells. The latter mechanism allows killing of myeloid APCs (18, 19, 25, 26). Additional TFs have been shown to activate *IL-10* promoter during IL-27-mediated induction of Tr1 cells: the early response gene 2 (27) and B lymphocyte-induced maturation protein-1 (Blimp-1) (28). Based on the above studies, it has been proposed that two transcriptional components activate *IL-10* in Tr1 cells upon IL-27 stimulation: c-Maf and Ahr are required for promoting IL-10 production under certain conditions, whereas Egr-2 *via* STAT3 induces Blimp-1 and IL-10 production (29). More recently, it has been suggested that after hematopoietic stem cell transplantation, Ag presentation in the presence of macrophage-derived IL-27 promotes Tr1 cell differentiation *via* Blimp-1 and eomesodermin (eomes). Eomes enables stable IL-10 production and consequently Tr1 cell induction *in vivo* (30). Moreover, the early induction of IRF1 and BAFT expression has been shown to be essential for IL-27-mediated induction of murine Tr1 cells (31). In the latter study, it was proposed that while BAFT is required for both Th17 and Tr1 cell induction, IRF4 and IRF1 are differentially required for the two cell subsets (31). However, this conclusion is in contrast with the demonstration that activin-A promotes human Tr1 cells *via* activation of IRF4 that along with AhR binds to *IL-10* and *icos* promoters (32). Moreover, ITK signaling *via* Ras/IRF4 pathway regulates the induction and function of both murine IL-27-induced Tr1 cells and human IL-10-induced Tr1 cells (33). ITK kinase activity is indeed critical for AhR, c-MAF, and IRF4 expression in T cells during Tr1 cell differentiation. Overall, despite the increased knowledge on the different pathways involved in promoting IL-10 production in T cells during Tr1 cell induction *via* IL-27 or IL-10, the master TF for Tr1 cells still remains unclear.

*In vivo* studies demonstrated that Tr1 cells circulate in peripheral blood (7) but are induced and also localized in tissues where IL-10 plays an essential role in maintaining homeostasis, such as the intestinal mucosa (34, 35). Recent observations in preclinical models indicate that Tr1 cells induced in the intestinal mucosa migrate to the periphery and control effector T cell responses and the development of type 1 diabetes (36). Interestingly, *in vitro* induced human Tr1 cells express the gut-homing receptors GPR15 and CCR9, supporting the capacity of Tr1 cells to migrate to the intestinal mucosa (37). Moreover, *in vivo* induced Tr1 cells have been identified in the spleen of tolerant mice (38). It still remains unclear whether Tr1 cells are generated in the spleen or if the spleen represents the *in vivo* natural “reservoir” of Tr1 cells. Moreover, it has not been demonstrated yet whether this organ is also the privileged site for Tr1 cell accumulation in humans. The discovery of CD49b and LAG-3 as specific biomarkers of Tr1 cells (7) renders possible to better study the *in vivo* localization of Tr1 cells in physiological and pathological conditions.

## Tr1 Cells and their Role in Tolerance Induction

A defect in Tr1 cell frequency/function has been consistently demonstrated in a number of autoimmune and inflammatory diseases in preclinical and clinical models, indicating that IL-10-producing Tr1 cells are relevant for disease protection [reviewed in Ref. (10)]. These evidences built the rationale for medical intervention for Tr1 cell boosting *in vivo* to prevent/cure T cell-mediated diseases. Several stimuli have been used to promote Tr1 cell induction *in vivo*. We and other demonstrated the ability of IL-10 or IL-10-inducing agents in combination with immunosuppressive treatments to generate Tr1 cell in *in vivo* models of autoimmunity or allogeneic transplantation. Among others, interesting treatments to promote Tr1 cells *in vivo* are the administration of anti-CD3 monoclonal antibodies or tolerogenic DCs, which in both preclinical models and in humans have been demonstrated to promote Tr1 cells. Alternatively, *in vivo* administration of soluble Ags has been proved to promote repolarization of autoimmune T cells into Tr1 cells in preclinical models [reviewed in Ref. (10)]. Some of these approaches have been translated to treat autoimmune diseases, including type 1 diabetes (T1D) and multiple sclerosis (MS). The first-in-man clinical trials with HLA-DR4-restricted proinsulin peptide or ATX-MS-1647, a cocktail of myelin basic protein-derived peptides, demonstrated the safety of these approaches without the induction/re-activation of pro-inflammatory autoimmune response, but with limited benefit for the patients (39, 40). Interestingly, in T1D patients, proinsulin peptide immunotherapy was associated with the transient appearance of Ag-specific IL-10^+^ CD4^+^ T cells (39), and in treated MS patients, a trend toward high levels of IL-10 gene expression associated with reduced Ag-specific T cell proliferation has been observed (40). These preliminary data indicate that peptide immunotherapy in autoimmune diseases may boost *in vivo* Ag-specific Tr1 cells.

T regulatory type 1 cells have been associated with long-term transplantation tolerance, induced or spontaneously established, in preclinical and clinical settings (38, 41, 42). Moreover, after the first demonstration that adoptive transfer of *in vitro* induced Ag-specific Tr1 cells efficiently prevents colitis induced in SCID mice by pathogenic T cells (9), several studies demonstrated that *in vitro* induced Tr1 cells can be used as cellular therapy to treat inflammatory and autoimmune disease as well as to control graft-versus-host disease (GvHD) and to prevent organ rejection [reviewed in Ref. (10)]. These evidences built the rationale for medical intervention with *in vitro* generated Tr1 cells to cure T-cell mediated diseases and to promote transplantation tolerance.

## Generation of Tr1 Cell Medicinal Products

**Figure 2:**
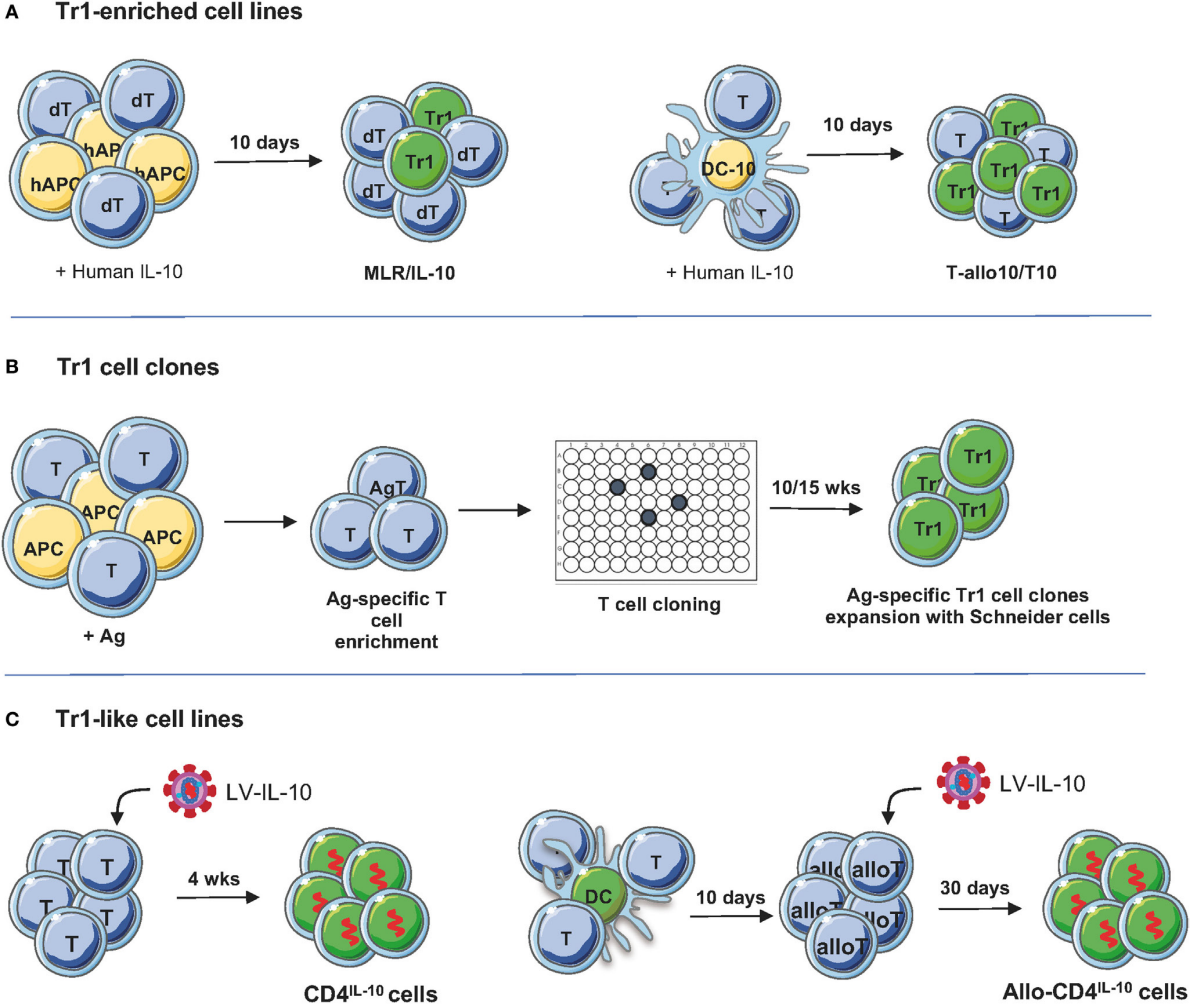
Protocols to generate/expand T regulatory type 1 (Tr1) cells. **(A)** Tr1-enriched cell lines. Donor-derived PBMC or CD4^+^ T cells are stimulated with host-derived monocytes in the presence of recombinant human IL-10 for 10 days. Alternatively, PBMC or CD4^+^ T cells are cultured for 10 days with allogenic dendritic cell (DC)-10, differentiated *in vitro* from peripheral blood monocytes with GM-CSF/IL-4/IL-10, in the presence of recombinant human IL-10 for 10 days (45). To generate T-allo10 cells, donor-derived T cells are cultured with host-derived DC-10 (Bacchetta and Roncarolo, unpublished data), whereas to induce host-derived T10 cells, T cells are stimulated with donor-derived DC-10 (46). **(B)** Tr1 cell clones. PBMC are stimulated with antigen (Ag; i.e., ovalbumin or collagen II) in the presence of IL-2 and IL-4 to enrich/expand Ag-specific T cells, followed by T cell cloning and expansion of the T cell clones using Schneider cells (4). **(C)** Tr1-like cell lines. Human CD4^+^ T cells are preactivated for 48 h with soluble anti-CD3/CD28 mAbs and IL-2 and then transduced with lentiviral vector (LV)-IL-10 overnight. Transduced T cells are isolated and expanded in feeder mixture (47). To generate allospecific IL-10-transduced cells, naive CD4^+^ T cells are stimulated with allogeneic DC and transduced with LV-IL-10 upon secondary stimulation. After selection, IL-10-trasduced cells are expanded *in vitro* with feeder mixture (48).

Several good manufacturing practice (GMP) compatible protocols have been established to generate human Ag-specific Tr1 cells. Originally, we induced alloAg-specific Tr1 cells by culturing human PBMCs (or purified CD4^+^ T cells) with allogeneic monocytes in the presence of exogenous human IL-10 [mixed lymphocytes reactions (MLR)/IL-10, Figure 2] (9). With this culture condition, a population of IL-10-anergized T cells is induced: these bulk populations are anergic in response to the alloAgs used for priming and contain precursors of IL-10-producing alloAg-specific Tr1 cells, as demonstrated by single cell T cell cloning of alloAg-specific Tr1 cells (9, 43). Moreover, after IL-10 anergization, the bulk cultures also contain non-alloAg-specific T cells that respond to other Ags, such as pathogens or third-party alloAgs (44, 45). Being a mixed population of cells, the IL-10-anergized cultures on one hand give rise to alloAg-specific Tr1 cells able to induce and sustain tolerance in the absence of immunosuppression, and on the other hand, they contain T cells with the ability to mount an efficacious immune response against infectious agents, when adoptively transferred into an immune-suppressed host. These features offer a strong rationale for the use of IL-10-anergized T cells as cell therapy to improve immunoreconstitution in immunocompromised hosts such as patients after allogeneic hematopoietic stem cell transplant (HSCT) and to modulate responses to alloAgs and promote long-lasting tolerance (Table 1).

**Table 1:** Tr1 cells in clinical and preclinical development.

Specificity	Name	Stage of development	References
**Tr1 cell products for clinical applications**			
Allospecific Tr1 cells	MLR/IL-10	ALT-TEN trial—completed	(49)
	T-allo10 cells	Phase 1 clinical trial—open now recruiting	T-allo10, NCT03198234
	T10 cells	GMP grade medicinal product	(46)
Ag-specific Tr1 cell clones	Ovasave^®^	CATS1 trial—completed	(50)
	Col-Treg	GMP grade medicinal product	http://www.txcell.com

**Tr1 cell products in preclinical development**			
Polyclonal Tr1-like cells	CD4^IL-10^ cells	*In vitro* and *in vivo* functional characterization	(47, 48)
Allospecific Tr1-like cells	Allo-CD4^IL-10^ cells	*In vitro* functional characterization	(48)

*GMP, good manufacturing practice; MLR, mixed lymphocytes reaction; Tr1, T regulatory type 1*.

The discovery of DC-10, a subset of monocyte-derived human DC, which secrete high levels of IL-10 and express the tolerogenic molecules ILT4 and HLA-G (51), offered the possibility to modify the protocol to generate alloAg-specific Tr1 cells (Figure 2). Functional assays demonstrated that stimulation of human PBMCs or CD4^+^ T cells with allogeneic DC-10 induces the differentiation of anergic alloAg-specific IL-10-producing Tr1 cells (45, 51). DC-10, in contrast to monocytes and exogenous IL-10, can promote T cell anergy not only in fully mismatched pairs but also in matched-unrelated pairs (45). Moreover, DC-10-anergized T cells contain up to 15% of already differentiated alloAg-specific CD49b^+^LAG-3^+^ Tr1 cells (Figure 2A, right), whereas, as discussed above, MLR/IL-10 cultures are enriched of IL-10-producing Tr1 cell precursors (Figure 2B) (43, 45). The MLR/IL-10 and MLR/DC-10 methods have been validated in GMP grade laboratories.

More recently, two optimized clinical grade compatible protocols for the induction of alloAg-specific Tr1 cells have been established. For a clinical trial in HSCT for hematological malignancies, a GMP compatible protocol in which purified donor-derived CD4^+^ T cells are cultured with tolerogenic DC-10 of host origin in the presence of IL-10 for 10 days to obtain alloAg-specific Tr1 cells (named T-allo10, Bacchetta and Roncarolo, Clinical-gov identifier NCT03198234) have been established (Table 1). In this setting, donor-derived T cells react against patient (host) alloAgs and mediate GvHD; therefore, to suppress GvHD after allo-HSCT, Tr1 cells *ex vivo* generated are donor-derived and specific for patient alloAgs. For a clinical trial in kidney transplant recipients planned under the umbrella of “The ONE study” (52), a GMP-compatible protocol to generate donor-specific Tr1-enriched cell medicinal product [named T10 cells (46)] has been developed (Table 1). In this protocol, donor-specific Tr1 cells are induced by culturing CD4^+^ T cells isolated from patients on dialysis with donor DC-10 in the presence of exogenous IL-10 for 10 days. In the contest of organ transplantation, patient T cells react against transplanted organ and mediate rejection; therefore, to prevent graft rejection, Tr1 cells *ex vivo* induced are patient-derived and specific for donor alloAgs. T10 and T-allo10 medicinal products contain a higher proportion of CD49b^+^LAG-3^+^ Tr1 cells compared to the IL-10-anergized T cells obtained by *in vitro* stimulation of donor-derived PBMCs with host CD3-depleted PBMCs in the presence of IL-10 [(46) and Bacchetta and Roncarolo, Clinical-gov identifier NCT03198234]. Tr1 cell medicinal products need to meet a number of release criteria for their *in vivo* delivery: (i) quality controls during the manufacturing, i.e., number, purity, and viability of CD14^+^ cells used for DC-10 differentiation, of CD4^+^ T cells, and of DC-10; (ii) quality controls of the final products, i.e., number, purity, and viability of T10 or T-allo10 cells, contamination of non-CD4^+^ T cells, and the percentage of allospecific T cell anergy, i.e., allospecific proliferation of T10 or T-allo10 medicinal product/allospecific proliferation of control cells generated in parallel by culturing CD4^+^ T cells with mature DC (46).

An alternative method to induce/expand Ag-specific Tr1 cell medicinal product has been developed by the France-based company TxCell.^1^ The method includes the use of *Drosophila*-derived artificial APCs (Schneider cells) transfected with a transmembrane form of a murine anti-human CD3 antibody, human CD80, human CD58, human IL-2, and human IL-4 (4). The expansion of Ag-specific Tr1 cell clones requires first stimulation of PBMCs with a specific Ag [i.e., ovalbumin (OVA)] in the presence of IL-2 and IL-4 to enrich for Ag-specific T cells, followed by T cell cloning by limiting dilution and expansion of the T cell clones using Schneider cells (Figure 1). This method has been applied to expand a large number of OVA-specific Tr1 cell clones (termed Ovasave^®^) that have been infused in patients affected with refractory Crohn’s disease (CD) (50) (Table 1). With a similar procedure, collagen II-specific Tr1 cell clones (Col-Treg) were produced from PBMCs of rheumatoid arthritis patients (5) (Table 1).

## Tr1-Based Clinical Trials

Treg cell-based cell therapy was first used to prevent GvHD after allogeneic HSCT. These proof-of-concept clinical trials demonstrated the feasibility and safety of the approach [reviewed in Ref. (53)] and paved the way to a wider application of Tregs as medical products for the treatment of autoimmune and chronic inflammatory disease and the prevention of organ rejection. Several trials are ongoing with different types of Tregs, including CD25^+^Tregs and Tr1 cells (53). A major difference between the CD25^+^ Tregs and Tr1 cells is that a pool of polyclonal non-Ag-specific cells are administered in the former, whereas Ag-specific products are infused with the latter.

T regulatory type 1 cell-based therapy mediated fast immune reconstitution and prevented severe GvHD in patients with advanced hematological malignancies undergoing haploidentical HSCT therapy [the ALT-TEN trial (49), Table 1]. A high dose (average of 12 × 10^6^/kg CD34^+^ cells) of haploidentical purified CD34^+^ hematopoietic stem cells, virtually devoid of T cells (≤2.6 × 10^4^/kg CD3^+^ cells), was infused in myeloablated patients. Once there were signs of myeloid engraftment, the donor-derived, host-alloAg-specific IL-10-anergized T cells were infused in patients in the absence of immunosuppression for GvHD prophylaxis. Results show that the treatment was safe and well tolerated. Clinical outcome of patients treated in the ALT-TEN trial suggests that donor-derived IL-10-anergized T cells could sustain immune reconstitution with no severe GvHD and no disease relapse. Moreover, the results suggest that a higher number of Tr1 cells within the total T cell suspension would provide enhanced activity to prevent GvHD. An improved method to generate alloAg-specific Tr1 cells (T-allo10 and T10 cells) using DC-10 has been established (see above). T-allo10 cells are currently tested in a phase I trial in patients with hematologic malignancies receiving un-manipulated HSCT from mismatched related or mismatched unrelated donors with the aim of preventing acute and chronic GvHD (T-allo10, Clinical-gov identifier NCT03198234, Table 1). In organ transplantation, as part of The ONE Study,^2^ T10 cells will be infused in living donor renal transplant recipients [(46), Table 1].

Tr1 cell-based therapy has been also tested to treat patients affected with refractory CD (50). In a phase I/IIa clinical, the CATS1 study, OVA-specific Tr1 cell clones (Ovasave^®^), generated as described above, were infused in CD patients, who ingested an OVA-enriched diet to activate OVA-specific Tr1 cells migrating to the gut (Table 1). Multiple doses of the cell product Ovasave^®^ were injected, and a response was observed in 40% of patients, with a stronger effect in the group of patients who received the lowest Tr1 cell dose (50). The study demonstrated the safety of the approach and showed some clinical benefit. However, the clinical effect was limited, reaching the maximum at 5 weeks after treatment and declining thereafter. Col-Treg will be tested in a clinical study for severe and refractory autoimmune uveitis (see text footnote 1; Table 1).

Overall these first clinical trials showed the feasibility, the safety, and potential clinical benefit of the Tr1 cell-based cell therapy approach. Because of their Ag specificity, Tr1 cells have the potential to be applied in several clinical settings.

## IL-10-Engineered CD4^+^ T Cells

The limitations to broaden the clinical application of Tr1 cells are as follows: (a) the presence of potential contaminating non-Tr1 cells in the preparation; (b) limited expansion capacity *in vitro*. The discovery of Tr1 cell-specific biomarkers, CD49b and LAG-3 (7), opened the possibility to isolate alloAg-specific Tr1 cells from *in vitro* cultures. Specifically, we showed that FACS-sorted CD49b^+^LAG-3^+^ T cells from DC-10-induced Tr1 cell populations had higher suppressive capacity compared to the original bulk population (7).

An alternative strategy to generate a large number of Tr1 cells is to induce stable and sustained overexpression of IL-10 into conventional CD4^+^ T cells. We developed a protocol based on the use of bidirectional LVs co-encoding for human *IL-10* and a marker gene of selection [i.e., GFP (47)]. We demonstrated that LV-mediated human *IL-10* gene transfer converted conventional human CD4^+^ T cells into Tr1-like cells, termed CD4^IL-10^ cells (Figure 2; Table 1). CD4^IL-10^ cells are phenotypically and functionally similar to Tr1 cells: they secrete high levels of IL-10, suppress T cell responses *via* IL-10 and TGF-β, and prevent xenoGVHD in humanized models (47). More recently, we modified our vector platform by substituting GFP with *ΔNGFR*, as a clinical grade marker gene for selection, and we demonstrated that CD4^IL-10^ cells acquire not only the potential to suppress T cell responses *in vitro* and *in vivo*, but also the ability to specifically kill myeloid leukemic cell lines and blasts in an HLA class I-dependent but Ag-independent manner (48). CD4^IL-10^ cells indeed selectively eliminate CD13^+^ leukemic cells, and for optimal killing of target cells, they require stable CD54/LFA-1-mediated adhesion and CD112/CD226-mediated activation. Importantly, CD4^IL-10^ cells mediate antitumor and antileukemic effects *in vivo* in humanized mouse models of solid myeloid tumors and leukemia (48). This newly identified antileukemic activity of CD4^IL-10^ cells is of critical importance, since an active area of investigation is the identification of regimens that prevents GvHD after allo-HSCT without affecting graft-versus-leukemia (GvL) activity of donor T lymphocytes. In a newly developed protocol of GvHD/GvL in humanized mice, we demonstrated that CD4^IL-10^ cells adoptively transferred *in vivo* prevent xenoGvHD mediated by allogeneic PBMC and collaborate with PBMC in mediating GvL. The LV-hIL-10 platform has been also applied to generate IL-10-engineered alloAg-specific Tr1-like cells, namely allo-CD4^IL-10^ cells (Figure 2; Table 1). Allo-CD4^IL-10^ cells suppress alloAg-specific T cell responses *in vitro* and kill myeloid target cells in an Ag-independent manner. Overall, we showed that enforced IL-10 expression in conventional or allospecific CD4^+^ T cells promotes their conversion into Tr1-like suppressor cells able to kill myeloid cell lines (48). These findings pave the way for adoptive cell therapy with IL-10-engineered T cells in patients undergoing allogeneic organ and HSCT transplantation for oncological diseases. Moreover, the antitumor and antileukemic activity of CD4^IL-10^ cells can be considered for *ad hoc* immunotherapy in relapsing patients.

## Future Perspectives

The discovery that Tr1 cells modulate immune responses led to the idea that they could be developed as a therapeutic product to promote/restore tolerance in transplantation and in inflammatory and autoimmune diseases. The completed clinical trials proved the safety of Tr1 cell-based therapy and indicate potential therapeutic effects. AlloAg-specific Tr1 cells can be generated *in vitro*, and protocols have been translated into clinical practice. It remained a major challenge to expand or generate Ag-specific Tr1 cells suitable for cell-based approaches in autoimmune diseases. Schneider cells have been shown to efficiently sustain Ag-specific Tr1 cell clone expansion for clinical application. However, Tr1 cell clones have limited survival capacity *in vivo* upon chronic activation (50, 54). The discovery that LV-mediated IL-10 gene transfer converts conventional polyclonal and alloAg-specific T cells into Tr1-like cells paves the way for applying this technology to generate a large number of Tr1 cells from Ag-specific T cells isolated from the peripheral blood of patients. Transcriptome analysis of this engineered Tr1-like cells will allow us to identify the key molecules involved in Tr1 cell immunomodulatory function.

## Author Contributions

SG and MGR designed and wrote the manuscript.

## Conflict of Interest Statement

The authors declare that the research was conducted in the absence of any commercial or financial relationships that could be construed as a potential conflict of interest.

## Footnotes

^1^http://www.txcell.com.

^2^http://www.onestudy.org/.
